# Faecal pollution affects abundance and diversity of aquatic microbial community in anthropo-zoogenically influenced lotic ecosystems

**DOI:** 10.1038/s41598-019-56058-x

**Published:** 2019-12-19

**Authors:** Lisa Paruch, Adam M. Paruch, Hans Geir Eiken, Roald Sørheim

**Affiliations:** Norwegian Institute of Bioeconomy Research (NIBIO), Division of Environment and Natural Resources, Fredrik A. Dahls vei 20, Oslo, 1433 Aas Norway

**Keywords:** Applied microbiology, Water microbiology, Biodiversity

## Abstract

The aquatic microbiota is known to be an important factor in the sustainability of the natural water ecosystems. However, the microbial community also might include pathogens, which result in very serious waterborne diseases in humans and animals. Faecal pollution is the major cause of these diseases. Therefore, it is of immense importance to assess the potential impact of faecal pollution, originating from both anthropogenic and zoogenic sources, on the profile of microbial communities in natural water environments. To this end, the microbial taxonomic diversity of lotic ecosystems in different regions of Norway, representing urban and rural areas, exposed to various levels of faecal pollution, was investigated over the course of a 1-year period. The highest microbial diversity was found in rural water that was the least faecally polluted, while the lowest was found in urban water with the highest faecal contamination. The overall diversity of the aquatic microbial community was significantly reduced in severely polluted water. In addition, the community compositions diverged between waters where the dominant pollution sources were of anthropogenic or zoogenic origin. The results provide new insight into the understanding of how faecal water contamination, specifically that of different origins, influences the microbial diversity of natural waters.

## Introduction

Surface water bodies constitute important sources of drinking water, estimated to cover approximately half of the worldwide need^1^. Yet, this overall coverage varies regionally, and in Norway, for instance, 85% of drinking water is supplied by surface fresh water sources. Unlike groundwater, which is protected by the overlying soils trapping and attenuating pollutants^2^, surface water bodies are more vulnerable to various contamination sites^1,2^. The polluted waters pose potential health risks to humans and the environment; thus, quality issues become the core concern for public and regulatory authorities. Water quality assessment considers physical, chemical, and biological aspects. However, considering the main health threats, the microbial profile represents the most important indicator of the biological quality of water environments^3–5^.

The aquatic microbiota contributes substantially to the sustainability (in terms of balanced physical, chemical and biological conditions, harmonised biodiversity and long-term functionality) of the natural (organic by nature) water ecosystems, playing key roles in organic and inorganic nutrient cycling, respiration, and biological and chemical contaminant removal in aquatic environments^6^. However, it can also pose a negative impact by carrying pathogens, which result in very serious waterborne diseases in both humans and animals^1,3^. Faecal pollution is the major cause of waterborne disease, since most of the pathogens associated with transmission reside in human and warm-blooded animal faeces. It is therefore unquestionable whether water contamination with enteric pathogens affects the overall quality of aquatic systems. The genuine question is how significant and to what extent it poses a threat to human and environmental health.

Faecal water contamination refers to a dual origin (anthropogenic and zoogenic) of various point and nonpoint/diffuse pollution sources (comprising discharge of industrial/municipal/domestic wastewater and storm/urban/agricultural water runoffs). It is normally determined by the detection and quantitation of viable bacterial indicators, so-called faecal indicator bacteria (FIB), represented primarily by *Escherichia coli* (*E. coli*) and certain *Enterococcus* species such as *E. faecalis* and *E. faecium*^7^. With respect to these FIB and the associated health aspects, various water quality standards and legislations have been established on both global and regional scales, e.g., drinking water guidelines^3,8^, bathing and recreational water criteria^9,10^, and management of alternative water resources^11^. These bacteria; however, cannot define either the origins or sources of the contamination, and therefore, a step forward in research efforts has been taken to identify the actual causes of faecal pollution^12,13^. This became accessible by implementing microbial source tracking (MST) methods using the most widely applied faecal bacterial source identifier – *Bacteroidales*^14^. They include bacteria that are one of the most abundant types in the intestine of humans and other warm-blooded animals. In particular, species of the genus *Bacteroides* comprise the major population of gastrointestinal microbes, and normally constitute approximately 30% of total faecal bacteria; although, this percentage can extend to over 50% of human faecal flora, with concentrations up to 10^11^ cells per gram of faeces^15^. Host-associated molecular markers derived from *Bacteroidales* 16S rRNA genes are by far the most tested/optimised via multiple molecular studies and have exhibited wide geographical stability^16–20^.

In recent decades, various molecular approaches have been developed to characterise microbial community structure in aquatic ecosystems^6^. Among these, DNA fingerprinting techniques have been one of the most frequently used; however, they are inadequate for taxonomic identification due to weak reproducibility of data and low resolution of community profile^21,22^. More recently, the advance of next-generation sequencing (NGS) technologies has enabled a deeper and better resolution of taxonomic characterisation; thus, both 454 pyrosequencing and Illumina MiSeq have been employed successfully for aquatic microbiota screening^23,24^. These new approaches using 16S rRNA and NGS provide significant insight into specific microbial taxonomic characterisation that otherwise is not apparent by traditional microbiological methods^22,25^.

Although the molecular techniques have proven powerful as tools for studying microbial activities in various water ecosystems, the composition of microbiota remains sparsely addressed in aquatic research^6^. Furthermore, very limited information is available regarding the diversity of water microbial communities impacted by faecal pollution. There are a few studies emphasising these issues, yet only the overall response of bacterial (no other microbes) diversity to anthropogenic (no other origin) faecal pollution is generally addressed^25,26^. In addition, most of the relevant studies refer to benthic microbiota in marine and lentic ecosystems, which is usually limited to a single domain, primarily *Bacteria*^22,23^. Hence, the real composition of microbial communities in lotic environments remains largely unknown^6,27^, and therefore a better picture of microbial diversity in such ecosystems is needed^22^.

In response to the aforementioned limitations, knowledge gaps, and needs, we applied Illumina MiSeq NGS of the 16S rRNA genes to characterise the microbial taxonomic diversity of faecally polluted lotic ecosystems in different regions of Norway. Faecal pollution is expected to influence the aquatic microbial community structure. Some might argue that faecal microbial communities are much more diverse than freshwater communities, thus higher diversity occurs in faecally polluted waters^28,29^. Others would argue that faecal contamination has a negative impact, thus reduce the overall aquatic microbial diversity^26,30,31^. These inconsistent opinions did motivate us to conceive and conduct this work in order to contribute with new insight into the understanding of how faecal pollution affects the microbial diversity of lotic waters. To the best of our knowledge, this is one of the pioneering studies dedicated solely to the application of NGS for the characterisation of aquatic microbial diversity associated with various levels and sources of faecal water contamination in Norway.

## Results and Discussion

### Faecal water contamination and its origin

**Figure 1:**
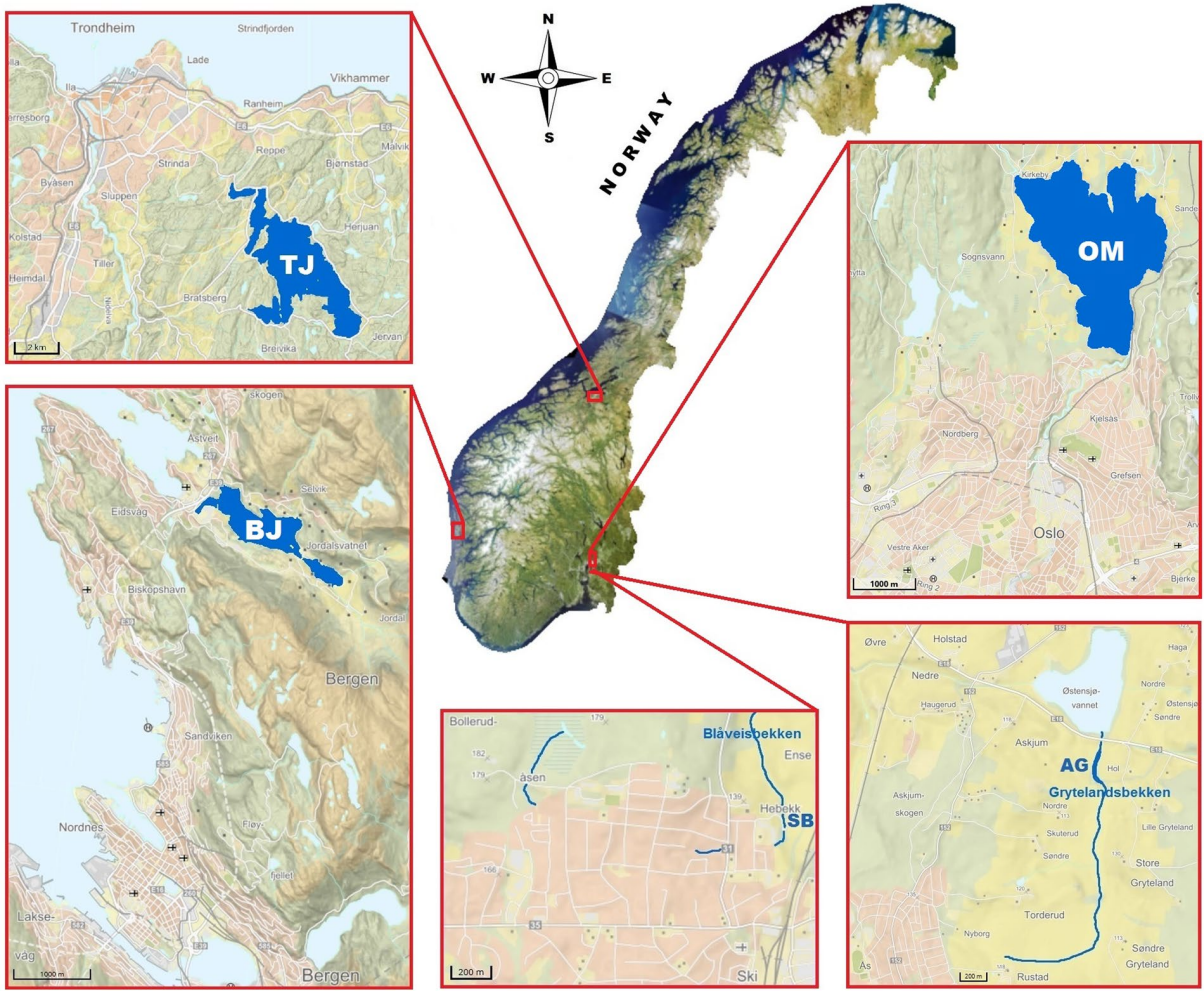
Location of the study sites: TJ – Trondheim, Jonsvannet drinking water reservoir; BJ – Bergen, Jordalsvatnet drinking water reservoir; OM – Oslo, Maridalsvannet drinking water reservoir; AG – Aas, Grytelandsbekken rural creek; and SB – Ski, Blåveisbekken urban stream. The satellite image and maps were obtained from NIBIO’s primary map service the Source/Kilden (https://kilden.nibio.no/?lang=nb&topic=arealinformasjon&bgLayer=norgeibilder_cache2&X=7334000.00&Y=400000.00&zoom=0, https://kilden.nibio.no/?lang=nb&topic=arealinformasjon&bgLayer=farger_cache&X=7037946.92&Y=275666.55&zoom=7#).

The selected study sites (a rural creek – AG, an urban stream – SB, and tributaries of drinking water reservoirs – BJ, OM and TJ, Fig. 1) revealed constant exposure to faecal pollution. Therefore, following the proposed screening criteria (described in “Materials and Methods” section), we investigated samples with an extreme variation in *Escherichia coli* (*E. coli*) concentrations (lowest vs. highest) and those with divergent origins of faecal contamination. The origin was expressed as the percentage contribution profile of host-specific *Bacteroidales* 16S rRNA genetic markers (AllBac, BacH, BacR and Hor-Bac) in the measured faecal contamination. These particular outcomes will not be addressed herein, as they have been described in greater detail elsewhere (detailed results of these examinations are available through open access reports published online at http://hdl.handle.net/11250/2392445, http://hdl.handle.net/11250/2392629, and http://hdl.handle.net/11250/2392631).

The highest *E. coli* numbers were found in the urban stream (SB), while the lowest were detected in the rural creek (AG). Furthermore, the contribution profile of the tested *Bacteroidales* DNA markers exposed the dominant anthropogenic origin in SB vs. zoogenic origin in AG (Table 1). This responded accurately to the divergent type of these two watercourses. The tributaries of drinking water reservoirs (OM, TJ, and BJ) also revealed relatively high *E. coli* numbers. The contribution profile of the tested genetic markers in faecal water contamination exposed the dominant zoogenic origin at the BJ and TJ sites, and exclusive zoogenic origin (100%) at the OM site (Table 1).

**Table 1:** Concentrations of *Escherichia coli* (*E. coli*) expressed as the most probable number (MPN) in 100 ml of water sampled in the study sites (AG – Aas, Grytelandsbekken rural creek; SB – Ski, Blåveisbekken urban stream; BJ – Bergen, Jordalsvatnet drinking water reservoir; OM – Oslo, Maridalsvannet drinking water reservoir; and TJ – Trondheim, Jonsvannet drinking water reservoir). Number of samples with the dominant anthropogenic or zoogenic faecal contamination in water.

Study sites	*E. coli* counts min – max mean	Dominant origin of faecal contamination in water samples		Total number of tested samples
		anthropogenic	zoogenic	
AG	1–144.5 9	1	9	10
SB	560–20 050 3 897	8	2	10
BJ	1–200.5 23	2	13	15
OM	2–200.5 38	0	10	10
TJ	2–200.5 58	2	13	15

### Microbial community composition and cross-comparison

In the microbial diversity study, 13,167,241 sequence reads were extracted and obtained following pair-end reads assembly. They were evenly distributed among all the examined samples. After quality filtering, 615,567 unique reads, with an average length of 265 bp, were used in downstream analysis. A total of 49,107 operational taxonomic units (OTUs) were predicted, with 32,272 assigned to bacteria, 1,722 to archaea, and the remaining 15,113 were uncharacterised. The taxonomic classification after assigning the clustered OTUs against the Greengenes Database (at 97% identity level), 74 phyla, consisting of 71 bacteria (96%) and 3 archaea (4%), were characterised, and a further 1,354 genera were identified.

**Figure 2:**
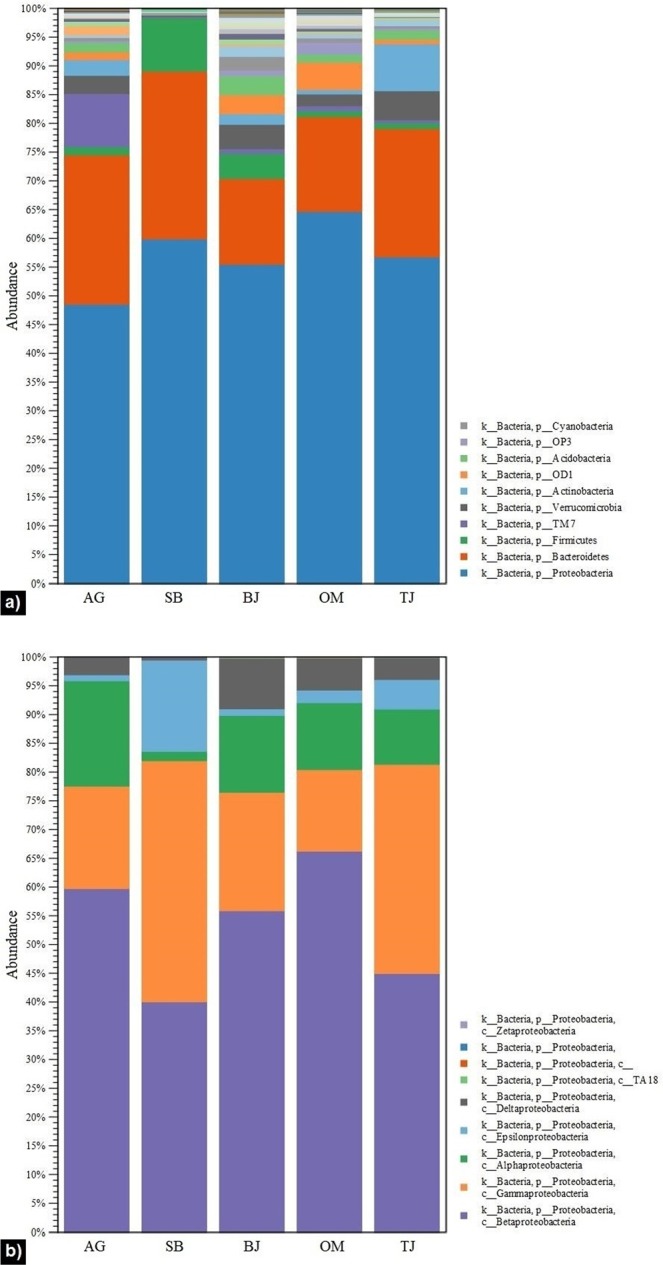
Relative abundance of bacterial populations among all studied sites (AG – Aas, Grytelandsbekken; SB – Ski, Blåveisbekken; BJ – Bergen, Jordalsvatnet; OM – Oslo, Maridalsvannet; and TJ – Trondheim, Jonsvannet): (**a**) all bacterial phyla, (**b**) *Proteobacteria* class distribution. The stacked bar charts were generated using the tools in CLC Microbial Genomics Module version 2.5.1 (CLC Bio, QIAGEN Company, Aarhus, Denmark, https://www.qiagenbioinformatics.com/clc-microbial-genomics-module-latest-improvements).

**Figure 3:**
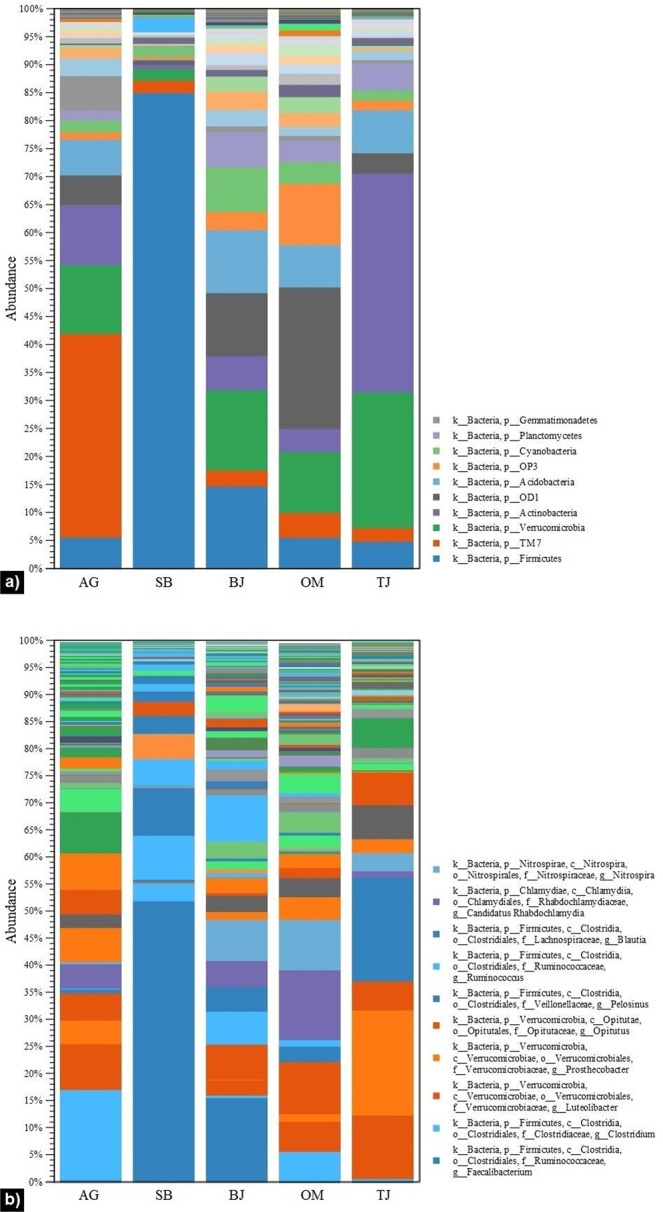
Relative abundance of bacterial populations among all studied sites (AG – Aas, Grytelandsbekken; SB – Ski, Blåveisbekken; BJ – Bergen, Jordalsvatnet; OM – Oslo, Maridalsvannet; and TJ – Trondheim, Jonsvannet): (**a**) bacterial phyla other than *Proteobacteria* and *Bacteroidetes*; (**b**) genera distribution of the other bacterial phyla. The stacked bar charts were generated using the tools in CLC Microbial Genomics Module version 2.5.1 (CLC Bio, QIAGEN Company, Aarhus, Denmark, https://www.qiagenbioinformatics.com/clc-microbial-genomics-module-latest-improvements).

Bacterial population distribution over the studied lotic ecosystems in different regions of Norway is presented in Figs. 2 and 3. The sequencing data analyses revealed that *Proteobacteria* and *Bacteroidetes* were two predominant phyla, representing 70–90% relative abundance (Fig. 2a). Published findings have reported that these phyla are “the core” bacterial population in lotic waters^20,26^. Furthermore, our outcomes indicate that *Proteobacteria* contributed mostly (from over 48% to nearly 65%) to the total OTU numbers. They were represented by nine classes (Fig. 2b), but primarily by *Betaproteobacteria*, *Gammaproteobacteria*, and *Alphaproteobacteria*, which is consistent with discoveries from previous relevant studies^32,33^. The class distribution pattern was very similar in all tested waters, with the exception of site SB (Fig. 2b), which harboured relatively low amounts of *Alphaproteobacteria* (2%), but higher amounts of *Epsilonproteobacteria* (16%). After filtering out these two predominant phyla, we also detected prevalence of other phyla, such as *Firmicutes*, *TM7*, *Verrucomicrobia*, *Actinobacteri*a, and *Acidobacteria* (Fig. 3a). For instance, *TM7*, *Verrucomicrobia*, and *Actinobacteri*a were three top phyla at site AG; *Firmicutes*, *Verrucomicrobia*, and *Acidobacteria* at site BJ; *OD1*, *OP3*, and *Verrucomicrobia* at site OM; and *Actinobacteria*, *Verrucomicrobia*, and *Acidobacteria* at site TJ. A notable observation was that *Firmicutes* bacteria were found at a remarkably high proportion (85% relative abundance) in the urban stream (site SB with the dominant anthropogenic faecal contamination in water). Furthermore, 52% of this phylum was assigned to the genus *Faecalibacterium* (Fig. 3b). While *Firmicutes* constitute the largest portion of the human gastrointestinal microbiome^34,35^, *Faecalibacterium* is one of the most abundant and important commensal bacteria in the human gut microbiota^36^. The importance of this observation is that it demonstrates a strong relationship and agreement with the results from faecal source tracking using the genetic markers in quantitative PCR, which showed a high dominance of human faecal contamination in the urban stream (Table 1). Very relevant to our outcomes are the findings by Unno *et al*.^37^ reporting *Firmicutes* as one of the major human faecal bacteria found in watersheds. Similar discoveries were recently revealed in a study by Sun *et al*.^29^, who detected high levels of *Firmicutes* in the Yangtze River and attributed them to the faecal bacteria carried in the effluent discharged from a nearby wastewater treatment plant.

**Figure 4:**
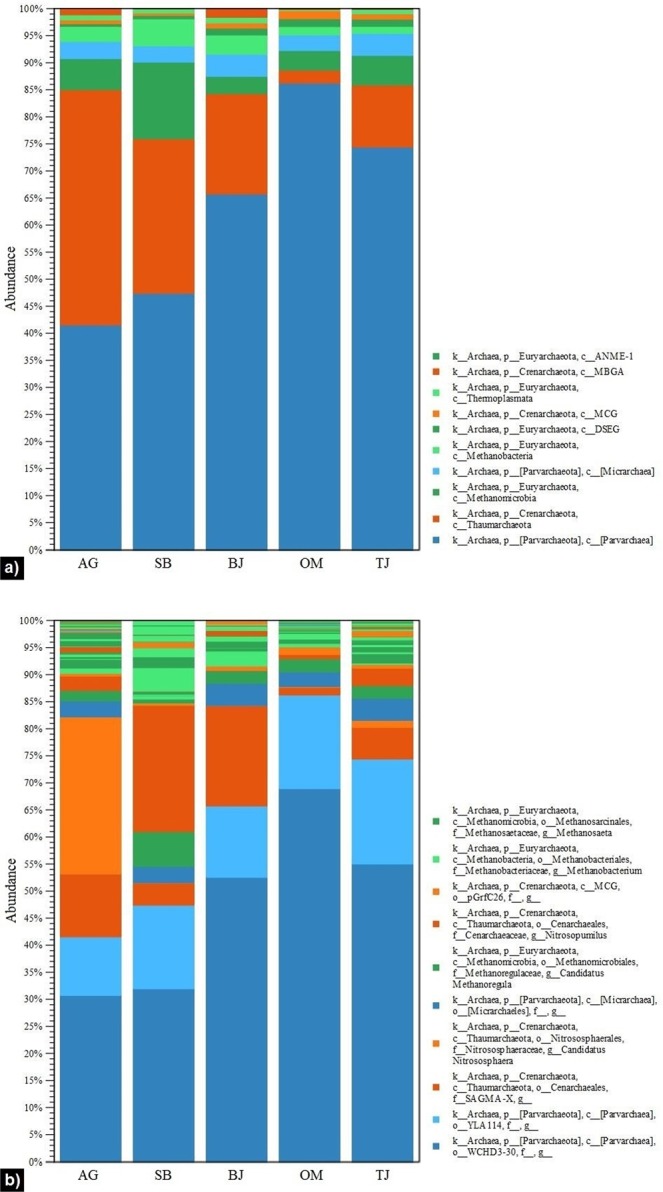
Relative abundance of archaeal populations among all studied sites (AG – Aas, Grytelandsbekken; SB – Ski, Blåveisbekken; BJ – Bergen, Jordalsvatnet; OM – Oslo, Maridalsvannet; and TJ – Trondheim, Jonsvannet): (**a**) phylum and class level; (**b**) genus level. The stacked bar charts were generated using the tools in CLC Microbial Genomics Module version 2.5.1 (CLC Bio, QIAGEN Company, Aarhus, Denmark, https://www.qiagenbioinformatics.com/clc-microbial-genomics-module-latest-improvements).

With regards to the archaeal population, all sequences were assigned to three phyla: *Euryarchaeota*, *Crenarchaeota*, and *Parvarchaeota*, with predominance of the latter (Fig. 4a). The absolute dominance of *Parvarchaeota* (65–86% relative abundance) was revealed in the tributaries of drinking water reservoirs (BJ, OM, and TJ). *Crenarchaeota* composed the second largest phylum, especially in water from sites AG (44%) and SB (30%), dominated by two particular genera, *Candidatus* Nitrososphaera and *Nitrosopumilus*, respectively (Fig. 4b). The latter represents an active archaeon that oxidises ammonia to nitrite, while the former was firstly sequenced from soil and was further proven to be positively correlated with agricultural practices and management such as fertilization with nitrogen compounds and farmyard manure^38^. This greatly links to the type of our study site, the rural creek (AG) in an agriculturally dominated catchment, which consists largely of farmlands (60%) and forest/marshlands (31%). Furthermore, the seasonal quantitative microbial source tracking (QMST) investigations conducted at this study site exposed agricultural run-off to be the major zoogenic source contributing to faecal water pollution^17^.

**Figure 5:**
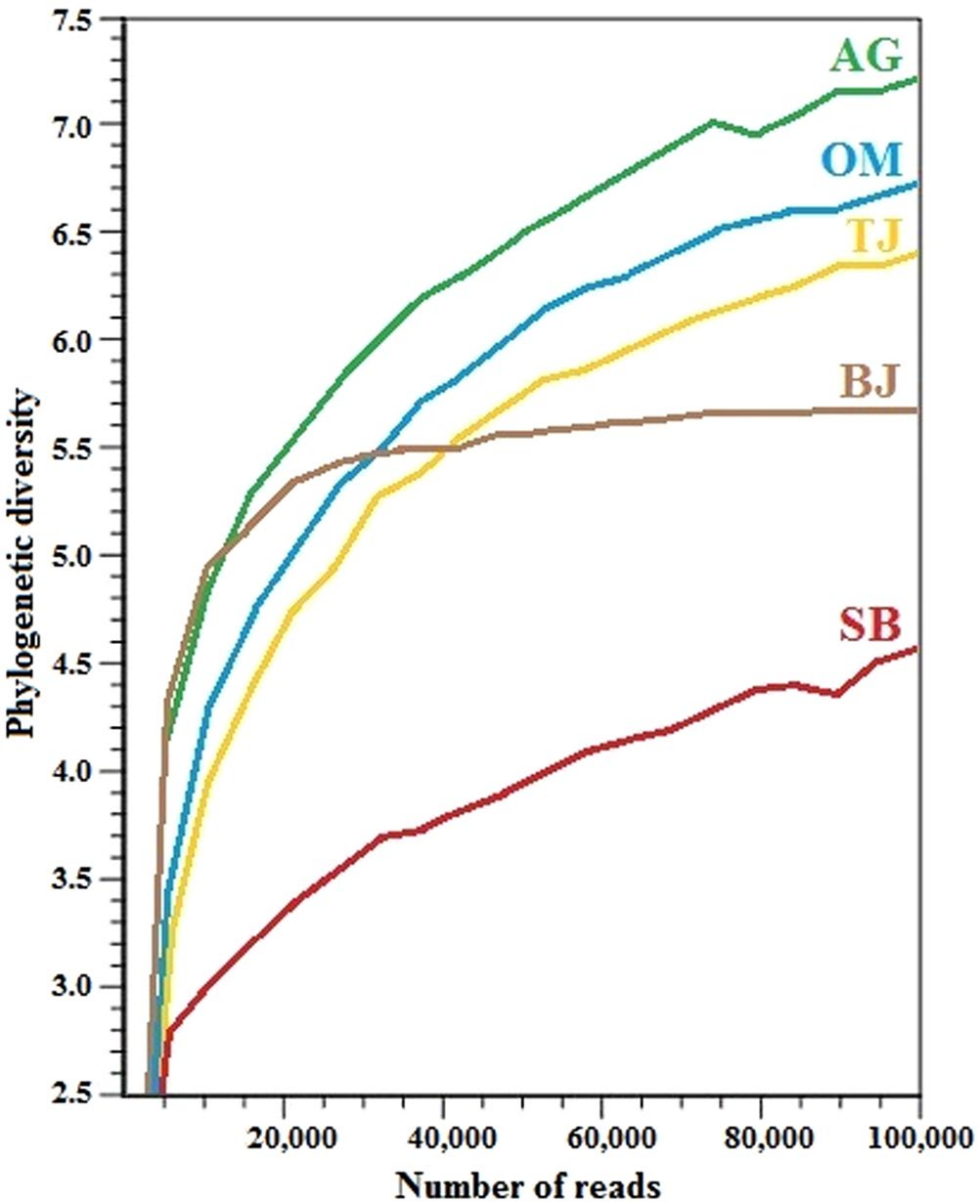
Phylogenetic alpha diversity of all studied sites (AG – Aas, Grytelandsbekken; SB – Ski, Blåveisbekken; BJ – Bergen, Jordalsvatnet; OM – Oslo, Maridalsvannet; and TJ – Trondheim, Jonsvannet). The alpha rarefaction plot was generated using the tools in CLC Microbial Genomics Module version 2.5.1 (CLC Bio, QIAGEN Company, Aarhus, Denmark, https://www.qiagenbioinformatics.com/clc-microbial-genomics-module-latest-improvements).

The alpha diversity depicted in the plot as a function of rarefaction estimation (Fig. 5) indicates that the diversity and richness profiles were relatively variable from site to site. As a result, the complete alpha diversity had the following order: AG > OM > TJ > BJ > SB. The highest diversity was found in the least polluted rural creek (site AG with the dominant zoogenic faecal contamination in water), while the lowest was exposed in the urban stream (site SB), which suffered from an extreme anthropogenic faecal contamination (Table 1). This indicates that faecal pollution has a negative impact on the aquatic microbial community structure. It is presumably attributed to the fact that faecal input constitutes an extra selective pressure on aquatic microbial community, in a way to selectively promote the enrichment of some favourable microbes and thus result in the suppression of other microbial populations. This responds to the fact that any discharge of faecal matter contributes to the increased load of chemical, biological, and organic contaminants in water environments. These further create various stress factors on the basic ecological processes of ecosystems, such as disturbance in water and energy cycle, nutrient flow and availability, and biodegradation. In response to such stresses, the microbial community will be expectedly re-shaped. Such changes have been reported in association with nutrient and organic matter variabilities^39,40^ as well as variations in temperature, oxygen, salinity, and acidification^26,41^. Significant impacts of combined various organic and inorganic contaminates on the microbial community structure in aquatic ecosystems have also been observed^42,43^.

**Figure 6:**
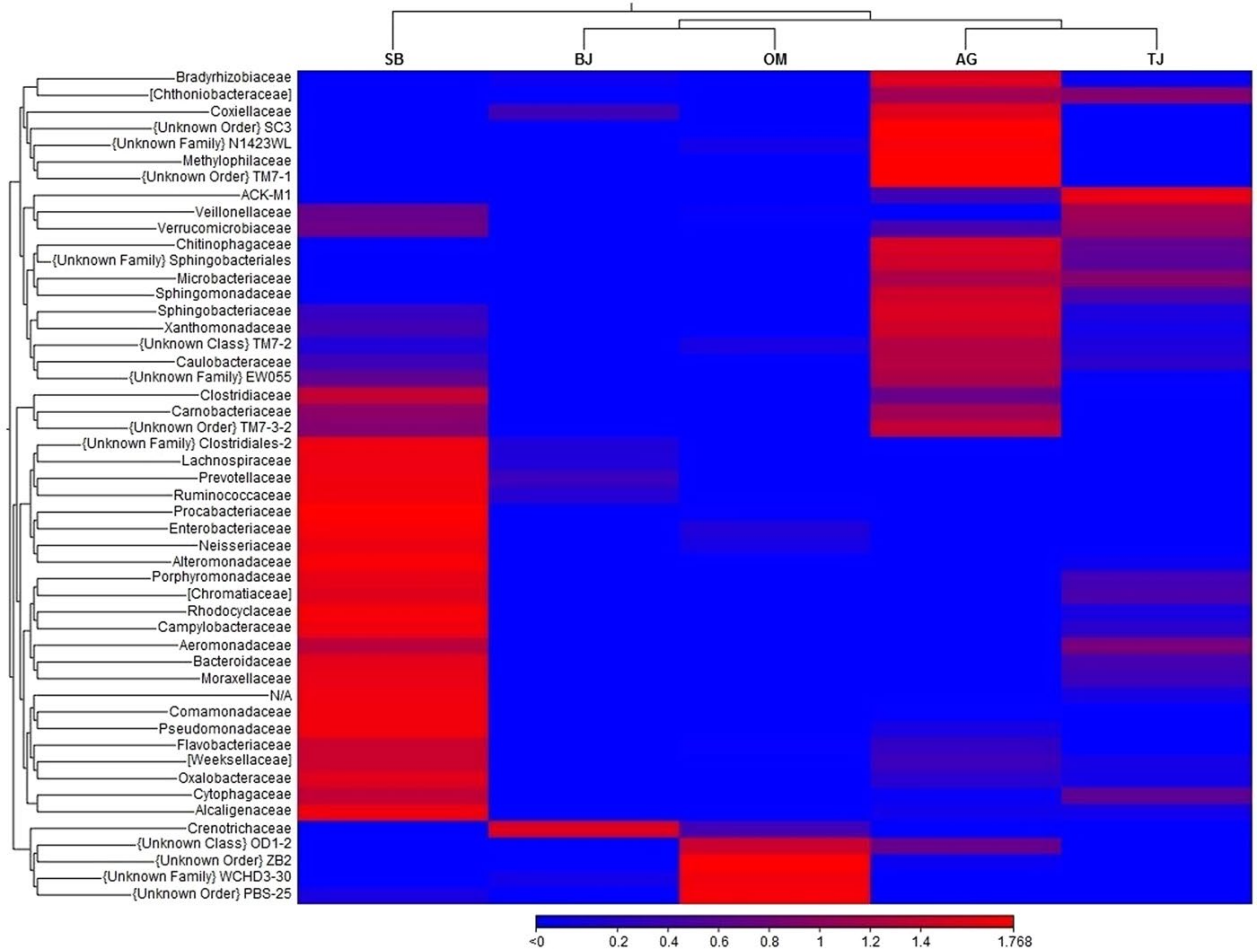
Beta diversity heat map with hierarchical clustering of the study sites (AG – Aas, Grytelandsbekken; SB – Ski, Blåveisbekken; BJ – Bergen, Jordalsvatnet; OM – Oslo, Maridalsvannet; and TJ – Trondheim, Jonsvannet). The hierarchical clustering heat map was generated using the tools in CLC Microbial Genomics Module version 2.5.1 (CLC Bio, QIAGEN Company, Aarhus, Denmark, https://www.qiagenbioinformatics.com/clc-microbial-genomics-module-latest-improvements).

**Figure 7:**
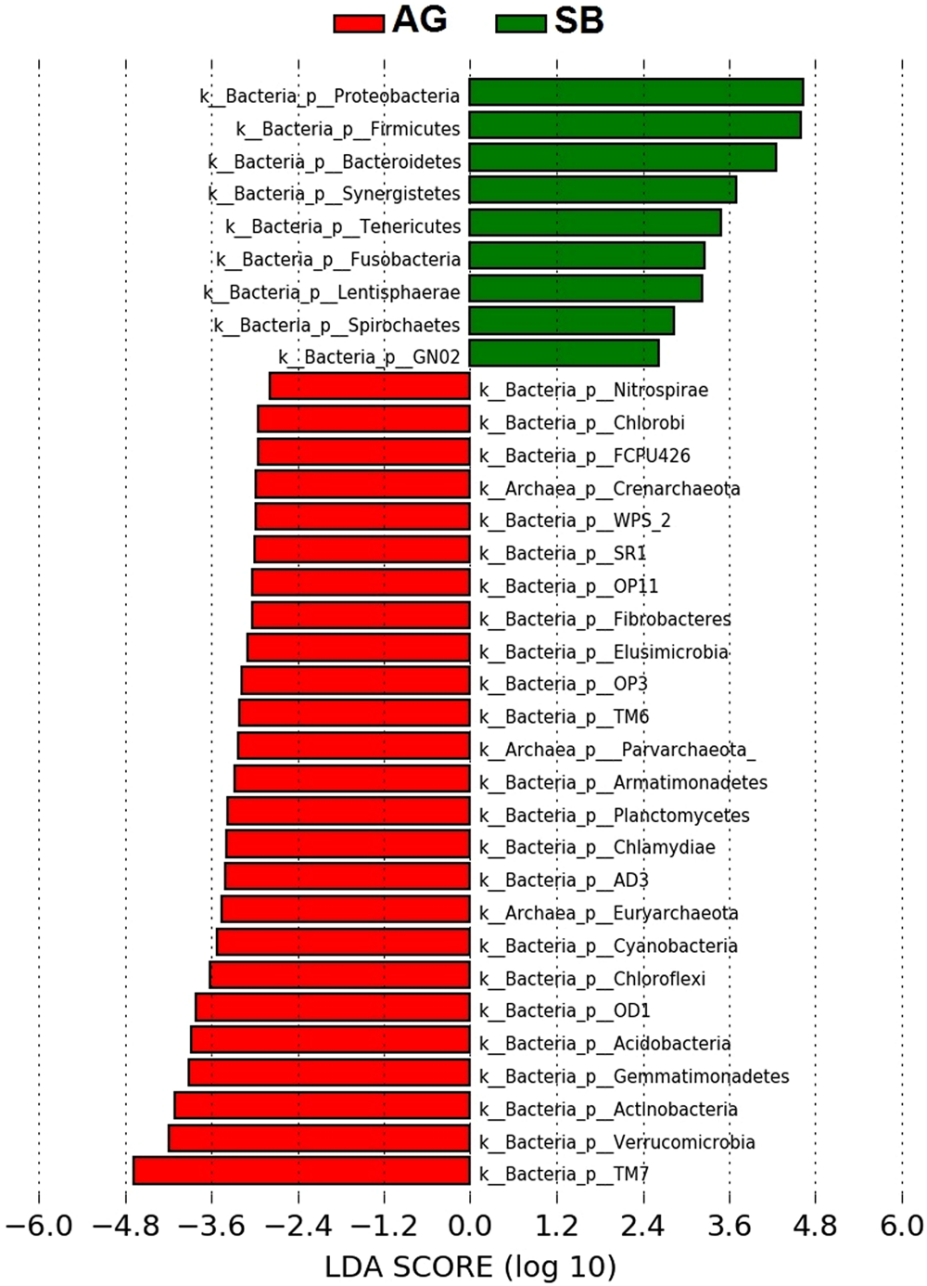
Linear discriminant analysis Effect Size (LEfSe) rank plot of differentially abundant microbial taxa in the least and most faecally polluted sites (AG – Aas, Grytelandsbekken and SB – Ski, Blåveisbekken, respectively) expressed as Linear Discriminant Analysis (LDA) logarithmic scores. The LEfSe plot was generated using the tools in Galaxy Version 1.0 (the Huttenhower Lab, Harvard School of Public Health, Boston, MA, USA, http://huttenhower.sph.harvard.edu/galaxy).

The beta diversity heat map (Fig. 6) visualises the hierarchical clustering of the sites based on their similarities of microbial compositions. PERMANOVA analysis reveals that the different clusters based on the groups were statistically significant (*p*-value = 0.00097). In this hierarchy, the two most-related clusters were disclosed, AG–TJ and BJ–OM. These were further merged to compose a separate cluster with the single site SB. This analysis clearly reveals that microbiota in the urban stream (SB) exposed to extremely high anthropogenic faecal pollution was greatly deviated from that found at the other study sites with the dominant zoogenic faecal contamination in water. Furthermore, the LEfSe algorithm (Fig. 7) carried out on the characterised microbial phyla in the divergent types of watercourses (rural/zoogenic – AG and urban/anthropogenic – SB) determined two distinct groups of microbes, which were apparently driving the microbial discrepancy between these two lotic ecosystems. As many as 184 distinct genera were considered to be differentially altered between the zoogenically and anthropogenically polluted waters. Moreover, the Wilcoxon rank-sum test reveals that the differences in microbial diversity between these two ecosystems were extremely significant (*p*-value < 0.0001). Typical human gut microbiota, mostly *Proteobacteria* (e.g. *E. coli*), *Firmicutes* (e.g. *E. faecalis* and *E. faecium*) and *Bacteroidetes* (e.g. *Bacteroidales*)^34,44^, was found more dominantly in the urban stream (site SB with the dominant anthropogenic faecal contamination in water), while in the rural creek (site AG exposed to pollution from zoogenic sources), more diverse microbes co-existed (Fig. 7), and many of them were often detected in natural waters, for instance, *Verrucomicrobia*, *SR1*, *OD1*, *OP11*, *OP3*, and *Planctomycetes*^45,46^.

Intriguingly, the statistical tests strengthen the analytical investigations by demonstrating practical examples of how different molecular methods could be used to interpret results, and thus validate the final conclusions. MST assays based on host-specific genetic markers have been widely implemented as a rapid and robust molecular method to identify the faecal origins in water. The assays are normally designed to target a specific group of faecal bacteria, for instance *Bacteroides*^47^, *Bifidobacterium*^48^, and *Faecalibacterium*^49^, which are genetically discriminated between the habitat hosts. Based on the generated quantitative data from QMST, a pollution-contributing profile can be further achieved^50^. In comparison, high-throughput sequence data acquired by NGS, the MiSeq technique in the present study, provide a comprehensive picture of the entire microbial community in water, and thus vary radically from the QMST by presenting total aquatic microbiota vs. target faecal species.

## Concluding Remarks

The joint application of QMST and NGS technologies in water faecal pollution research presents relatively comprehensive and mutually supportive approaches. The sequencing data provide extremely valuable input to better interpret the microbial community structure in the studied aquatic ecosystems, in particular, enabling closer monitoring of the alteration in microbial composition under various stresses, e.g. faecal pollution pressures. Findings derived from these technologies applied in our studies clearly reveal (analytically and statistically) that faecal pollution has a significant impact on the overall aquatic microbial community. The diversity of microbiota was substantially reduced in severely polluted water. Furthermore, the community compositions and richness profiles diverged greatly between lotic ecosystems, where the dominant faecal contamination sources were of anthropogenic or zoogenic origin. Consequently, water with high anthropogenic faecal pollution demonstrated low diversity of microbiota, while more distinct aquatic microbial profile characterised ecosystems exposed to zoogenic faecal sources.

## Materials and Methods

### Data screening for sampling site selection

To select the most distinctive sites characterising the microbial taxonomic diversity of faecally polluted lotic ecosystems in Norway, we focused on choosing those that accomplished most of the following criteria: (1) continuously exposed to varying pressures of faecal contamination, but not from direct input sources (e.g. direct discharges of sewage or effluent from wastewater treatment plant); (2) characterise sensitive spots (e.g., drinking water sources); (3) represent different geographical regions of the country; and (4) include dissimilar types of watercourses. For these purposes, we conducted site screening based on the original data from our recent studies assessing the rates of faecal pollution in tributaries of drinking water reservoirs supplying water to Norway’s three largest cities: Oslo (680 000 citizens), Bergen (280 000 citizens), and Trondheim (190 000 citizens) located in different regions of the country (Eastern, Western, and Central Norway; Fig. 1). In addition, we utilised data of microbial quality measures carried out in two different types of watercourses (an urban stream partially culverted and a rural creek) located in the neighbouring municipalities, Ski (30 000 people) and Aas (20 000 people), located 20 and 30 km south-east of Oslo, respectively (Fig. 1).

Consequently, all the analysed data were derived from our latest national projects conducted in cooperation with local authorities/municipalities of Oslo, Bergen, and Trondheim, and partly from an international project, *AQUARIUS* (https://eeagrants.org/project-portal/project/CZ09-0013). Based on the preliminary screening of the numerous data, we selected the most representative sampling sites exposed to the constant pressure of faecal pollution originating from various anthropogenic and zoogenic sources. These were confirmed by standard *Escherichia coli* (*E. coli*) examinations and quantitative microbial source tracking (QMST) using host-associated genetic markers, i.e. AllBac (universal marker), BacH (human), BacR (ruminant) and Hor-Bac (horse). Detailed results of these examinations are available through open access reports published online at http://hdl.handle.net/11250/2392445, http://hdl.handle.net/11250/2392629, and http://hdl.handle.net/11250/2392631. Scientific backgrounds and methodological procedures have been described in greater detail elsewhere^50^.

In total, 60 water samples collected over the course of a 1-year period were examined (Table 1). The samples reflect an assortment of the site screening criteria, and thus represent lotic ecosystems with two extreme levels of faecal water pollution: the least contaminated site – a rural creek in Aas, named Grytelandsbekken (AG), and the most contaminated site – an urban stream in Ski, named Blåveisbekken (SB); as well as three sensitive sites located in different regions of Norway, i.e., tributaries of drinking water reservoirs for Oslo, Bergen, and Trondheim, named Maridalsvannet (OM), Jordalsvatnet (BJ), and Jonsvannet (TJ), respectively (Fig. 1). No specific background data on the sampled sites were collected, except the most divergent sites, i.e. AG (the least faecally polluted rural creek with the dominant zoogenic contamination in water) and SB (the site exposed to extremely high anthropogenic faecal pollution). In these two lotic ecosystems, physico-chemical parameters were measured at each (the same) sampling occasion (over the course of a 1-year period) and the data are presented in Table 2. Neither temporal and spatial features nor climatic conditions differentiated these two sites (Fig. 1), and therefore other external influences than the defined faecal water contamination were not substantial. For that reason, site AG and SB were deliberately selected for exploration of zoogenic vs. anthropogenic influences as well as the highest vs. lowest faecal contamination effects on the lotic microbial diversity, which is the new and compelling aspect of our research.

**Table 2:** Physico-chemical parameters measured in the least faecally contaminated site (AG – Aas, Grytelandsbekken rural creek) and the most faecally contaminated site (SB – Ski, Blåveisbekken urban stream). Values in mg/l, and mS/m for EC.

Parameter	Values min – max mean	
	AG	SB
Chemical oxygen demand (COD)	9–38 24	6–129 28
Total suspended solids (TSS)	5.6–92.3 17.7	6.1–1350 168
Total dissolved solids (TDS)	107–225 150	120–316 177
Total organic carbon (TOC)	3.1–12.4 7.6	3–9 5.9
Dissolved organic carbon (DOC)	3–12 7	3–8.4 5.6
Total phosphorus (TP)	0.017–0.228 0.083	0.028–0.71 0.114
Phosphate phosphorus (PO_4_-P)	0.013–0.094 0.032	0.004–0.112 0.036
Total nitrogen (TN)	2.56–9.66 5.08	1.5–6.36 3.13
Ammonium nitrogen (NH_4_-N)	0.004–0.899 0.123	0.026–2.17 0.307
Nitrite nitrogen (NO_2_-N)	0.008–0.068 0.03	0.005–0.183 0.041
Nitrate nitrogen (NO_3_-N)	1.84–4.37 2.72	1.21–2.02 1.54
Electrical conductivity (EC)	11.8–31.6 19.6	18–40.7 27.1
pH	6.9–7.7	7.4–8.4

### Faecal contamination, source tracking, and origin profiling

Detection of faecal water contamination and definition of its origins, namely anthropogenic and zoogenic, was performed according to the methodological toolbox implemented and verified in Norway^50^. The completed procedure consists of three independent subsequent steps: (1) faecal pollution detection – examination of *E. coli* using the Colilert 18/Quanti-Tray^®^2000 method (IDEXX Laboratories Incorporated, Westbrook, Maine, USA) and bacteria enumeration as the most probable number (MPN)/100 mL; (2) faecal source tracking – detection and quantitation of host-specific *Bacteroidales* 16S rRNA genetic markers through real-time quantitative polymerase chain reaction (RT-qPCR); and (3) faecal origin profiling – characterisation of faecal source contribution in the actual sample tested. The key focus is given to the confirmed faecal contamination (step 1); thus, only samples positive for *E. coli* were processed in further molecular investigations (step 2). Since there are no significant correlations between *E. coli* bacteria and the *Bacteroidales* DNA markers, the faecal origin profile (step 3) was assessed as a percentage contribution of the markers defined in the contaminated water. The scientific background and techniques of this toolbox have been described in greater detail elsewhere^50^.

### DNA extraction, PCR, and MiSeq sequencing

From each faecally polluted sample, 200 mL water was concentrated in an ultrafiltration unit. Microbial genomic DNA was extracted from each yielded filter using a PowerSoil® DNA Isolation Kit (MO BIO Laboratories, Carlsbad, California, USA), following the protocol applied in earlier studies^50^. Purified DNA materials from the same sampling site were pooled together in equal amount and proceeded to library preparation of 16S rRNA amplicons. Such DNA pooling strategy has been revised and implemented in other published studies covering various ecosystems (soil and water) and different macro-/microorganism communities^33,51–53^. Five nanograms of each pooled genomic DNA was used in PCR to generate an amplicon library for each sample, according to the procedure instructions of the NEXTflex^TM^ 16S V4 Amplicon-Seq Kit 2.0 (Bioo Scientific Corporation, Austin, TX, USA). Briefly, the method consists of two-step PCR procedures. In the first PCR, the 16S rRNA V4 region was amplified using V4-specific primer sets; following PCR purification using AMPure XP Magnetic Beads (Agencourt Bioscience Corporation, Beverly, MA, USA), the clean-up product was subjected to the second PCR, where an Illumina adaptor overhang and unique 12-bp index were attached to the final product. Following purification using AMPure beads, the DNA concentration was measured by a Qubit^TM^ Fluorometer (Life Technologies, Eugene, OR, USA) using a Quant-IT^TM^ dsDNA HS Assay Kit (Invitrogen, Carlsbad, CA, USA). Amplicon products were normalised and equimolar pooled. The pooled library sample was analysed by 1% agarose gel electrophoresis and subsequently sequenced by an Illumina MiSeq sequencer at the Norwegian Sequencing Centre (Oslo, Norway) using a MiSeq Reagent Kit V3 (Illumina Inc., San Diego, CA, USA).

### Sequencing data analyses

The sequencing reads were analysed using the Microbial Genomics Module 2.0 added onto the CLC Genomic Workbench 10.1.1 (CLC Bio, QIAGEN Company, Aarhus, Denmark http://www.qiagenbioinformatics.com/products/clc-genomics-workbench). The entire analysis workflow was composed of a number of sequential and interconnected steps, consisting of quality filtration of the sequence data, operational taxonomic unit (OTU) clustering, and alpha and beta diversity studies basically using default parameter settings as configured in the workflow of the Microbial Genomics Module. Sequence trimming involved adapter trimming, quality trimming, and length trimming. The primer sequence was removed and the reads with a quality score lower than 20 were discarded. The maximum accepted number of ambiguous nucleotides was 2, and the length of the reads was fixed at 200–500 bp. Furthermore, chimeric sequences including singletons were detected and trashed. The remaining qualified unique reads were used for OTU clustering, which was performed by alignment to the Greengenes Database (Greengenes v_13_5) at 97% sequence similarity. A phylogenetic tree was subsequently constructed using a maximum likelihood approach based on a multiple sequence alignment (MSA) of the OTU sequences by the MUSCLE tool. This was further applied for alpha and beta diversity measures. The former, expressed as species richness in an ecosystem, was estimated from the rarefaction analysis using the resulting phylogenetic tree with a maximum sampling depth to 100,000 reads. The latter, which examines changes in species diversity between ecosystems, was measured using the Euclidean distance criterion. All sequence data were deposited at the NCBI Sequence Read Archive under accession number SRP133171, as part of BioProject PRJNA434659.

### Statistical analyses

A beta diversity hierarchical clustering heat map was created to visualise the relatedness of the aquatic microbial ecosystems by the similarity of their communities. It was generated through the abundance of features in the tested samples and expressed as a binary tree clustering the study sites based on the Euclidean distance at the phylum level using the Trimmed Mean of M-values (TMM) and the Z-score (the number of standard deviations from the population mean) normalisations. In addition, PERMANOVA analysis was carried out to ascertain statistical significance of the clusters. These tests were executed by applying CLC Microbial Genomics Module version 2.5.1 (CLC Bio, QIAGEN Company, Aarhus, Denmark, https://www.qiagenbioinformatics.com/clc-microbial-genomics-module-latest-improvements). Moreover, a differential abundance analysis was performed using the Linear discriminant analysis Effect Size (LEfSe) algorithm^54^ to unravel/disclose the driving microbial groups, which were mostly responsible for the difference in the least and most faecally polluted ecosystems (AG and SB, respectively). The formatted OTU abundance table was uploaded to the Galaxy/Hutlab application web-based platform (Biostatistics Department, Harvard School of Public Health, Boston, MA, USA http://huttenhower.sph.harvard.edu/galaxy) for the pairwise comparison of the microbial taxa. The comparison was tested for statistical significance (the Wilcoxon rank-sum test) and the alpha value for the pairwise Wilcoxon test was set at 0.05. The identified features characterising the differences between the ecosystems were processed using Linear Discriminant Analysis (LDA) with a threshold score set at 2.0.

### Artwork

Figure presenting the locations of the study sites (Fig. 1) is based on a satellite image and topographical map of Norway provided by the Norwegian Institute of Bioeconomy Research (NIBIO). The Institute’s geographical data is recorded in the NIBIO’s primary map service the Source/Kilden (https://kilden.nibio.no). All maps and map images collected in the Source/Kilden are open for all and can be saved or printed (https://www.nibio.no/en/subjects/soil/national-land-resource-map?locationfilter=true).

Figures containing results and statistics (Figs. 2–7) were generated using the tools in CLC Microbial Genomics Module version 2.5.1 (CLC Bio, QIAGEN Company, Aarhus, Denmark, https://www.qiagenbioinformatics.com/clc-microbial-genomics-module-latest-improvements) and Galaxy Version 1.0 (the Huttenhower Lab, Harvard School of Public Health, Boston, MA, USA, http://huttenhower.sph.harvard.edu/galaxy).

## Data Availability

The datasets generated during the current study are available from the corresponding author on reasonable request.
